# Study of the sensitivity and specificity of smell and taste disorders as a predictive factor of SARS-CoV-2 infection among primary care healthcare professionals: a retrospective observational study

**DOI:** 10.3399/BJGPO.2021.0141

**Published:** 2022-05-18

**Authors:** Anna Ruiz-Comellas, Pere Roura Poch, Glòria Sauch Valmaña, Víctor Guadalupe-Fernández, Jacobo Mendioroz Peña, Queralt Miró Catalina, Josep Vidal-Alaball, Anna Ramirez-Morros

**Affiliations:** 1 Centre d'Atenció Primària (CAP) Sant Joan de Vilatorrada. Gerència Territorial de la Catalunya Central, Institut Català de la Salut, Sant Fruitós de Bages, Spain; 2 Health Promotion in Rural Areas Research Group, Institut Català de la Salut, Sant Fruitós de Bages, Spain; 3 Unitat de Suport a la Recerca de la Catalunya Central, Fundació Institut Universitari per a la Recerca a l’Atenció Primària de Salut Jordi Gol i Gurina, Barcelona, Spain; 4 Faculty of Medicine, Universitat de Vic–Universitat Central de Catalunya, Vic, Spain; 5 Consorci Sanitari de Vic, Hospital de Vic, Vic, Spain; 6 Epidemiological Surveillance and Response to Public Health Emergencies Service, Public Health Agency of Catalonia, Barcelona, Spain

**Keywords:** COVID-19, change in sense of taste, change in sense of smell, sensitivity and specificity, primary health care, general practice

## Abstract

**Background:**

Among the manifestations of COVID-19 are taste and smell disorders (TSDs).

**Aim:**

To evaluate the sensitivity and specificity of TSDs and other associated symptoms to estimate predictive values for determining SARS-CoV-2 infection.

**Design & setting:**

A retrospective observational study of healthcare professionals in Catalonia, Spain.

**Method:**

A study of the sensitivity and specificity of TSDs has been carried out using the polymerase chain reaction (PCR) test for the diagnosis of SARS-CoV-2 as the gold standard value. Logistic regressions adjusted for age and sex were performed to identify additional symptoms that might be associated with COVID-19.

**Results:**

The results are based on 226 healthcare workers with clinical symptoms suggestive of COVID-19, 116 with positive PCR and 110 with negative PCR. TSDs had an odds ratio (OR) of 12.4 (95% confidence interval [CI] = 6.3 to 26.2), sensitivity 60.3% and specificity 89.1%. In the logistic regression model, the association of TSD, fever or low-grade fever, shivering, dyspnoea, arthralgia, and myalgia obtained an area under the curve (AUC) of 85.7% (95% CI = 80.7 % to 90.7 %), sensitivity 82.8 %, specificity 80.0%, and positive predictive values 81.4% and negative 81.5%.

**Conclusion:**

TSDs are a strong predictor of COVID-19. The association of TSD, fever, low-grade fever or shivering, dyspnoea, arthralgia, and myalgia correctly predicts 85.7% of the results of the COVID-19 test.

## How this fits in

Initial Asian studies did not refer TSDs as a common symptom of COVID-19. TSDs seem to be a strong predictor of a positive COVID-19 result in the European population. The association of TSD, shivering, dyspnoea, arthralgia, and myalgia correctly predicted 85.7% of positive results of the COVID-19 test in Spanish patients with mild-to-moderate clinical symptoms. TSDs could be included as part of routine screening for COVID-19.

## Introduction

After severe acute respiratory syndrome caused by SARS-CoV coronavirus in 2002 and Middle East acute respiratory syndrome caused by MERS-CoV in 2012,^1^ another highly pathogenic coronavirus called SARS-CoV-2 appeared in December 2019 in Wuhan, China, and has spread rapidly around the world. On 11 March 2020, the World Health Organization (WHO) called this new coronavirus outbreak a pandemic.^2^ Currently this virus has infected more than 174 million people worldwide.^3^ SARS-CoV-2 is a single-stranded RNA (ssRNA), betacoronavirus, and the disease it causes is COVID-19.

According to initial clinical studies from Asia, the most common symptoms of COVID-19 were fever, cough, dyspnoea, myalgia, arthralgia, headache, odynophagia, and less frequently rhinorrhoea and nasal congestion.^4–6^ But as the disease has spread around the world, a more varied and complex clinical spectrum has been described. According to European data, there is increasing evidence that patients with COVID-19 may present with a heterogeneous spectrum. Many affected people with mild-to-moderate forms showed TSDs even without fever, cough, or other systemic abnormalities.^7–9^ It is common for many viruses, such as rhinoviruses, parainfluenza virus, and some coronaviruses^10,11^ to cause olfactory dysfunction through an inflammatory reaction of the nasal mucosa and the development of rhinorrhoea; however, the olfactory dysfunction associated with COVID-19 infection seems peculiar, as it is not associated with rhinorrhoea.

Initial studies identified fever, fatigue, cough, and dyspnoea as predictors of COVID-19.^12^ Subsequently, the TSDs were also associated with COVID-19.^7,13–15^

On observing clinical differences between populations, the aim of the study was to evaluate the sensitivity and specificity of TSDs and associated symptoms to estimate predictive values for SARS-CoV-2 infection in a sample of Spanish healthcare professionals from the central region of Catalonia, an autonomous community in north-western Spain.

## Method

A study of sensitivity and specificity of TSDs was designed using the reverse transcription-polymerase chain reaction (RT-PCR) test as the gold standard value, the diagnostic test for SARS-CoV-2 coronavirus.

A telephone interview was carried out with nurses and doctors in primary care of the Territorial Management of Central Catalonia (in the counties of Bages, Berguedà, Anoia, Solsonès, Moianès, and Osona) with clinical symptoms suggestive of COVID-19. Healthcare professionals were included with at least one positive PCR performed during the period from 10 March–10 April 2020, and who had presented with mild-to-moderate COVID-1 (defined as patients who did not require admission to intensive care units). Interviews were conducted between 4 May23 May 2020. There was a 95% response rate. The duration of the interview was 5–10 minutes. All data were extracted from the telephone interview.

Positive cases were considered to be those patients with at least one positive PCR for COVID-19 and negative cases were those patients with all negative PCRs. Exclusion criteria were patients who were asymptomatic but had a PCR because of close contact with a sick person; patients with olfactory or gustatory dysfunction before the epidemic; and patients hospitalised in the intensive care unit at the time of the study.

A total of 226 participants were included, randomly selected from a list of healthcare professionals symptomatic with flu-like symptoms or COVID-19 presumption provided by the technical unit of the Territorial Management of Central Catalonia of the Catalan Institute of Health. Of these, 116 were considered positive cases and 110 were considered negative cases.

The following sociodemographic variables were studied: age; sex; and professional category. The clinical variables were: previous changes in sense of taste and/or smell; smoking (does not smoke, smokes [number of cigarettes]); allergies (only environmental allergies such as to pollen, mites, and animal hair were considered); hospital admission; number of admission days; and laboratory results (PCR). For each clinical symptom it was asked whether it had occurred (yes/no) and for how long. The following were studied: fever (temperature ≥38°C); low-grade fever (temperature 37°C–37.9°C); cough; asthenia; anorexia; diarrhoea; odynophagia; abdominal pain; vomiting and/or nausea; dyspnoea; chest pain; shivering; conjunctival hyperaemia; lacrimation; dry eyes; blurred vision; sneezing; rhinorrhoea; nasal congestion and/or obstruction; epistaxis; tinnitus; hearing loss; sputum production; haemoptysis; tachycardia; headache; dizziness; impaired consciousness; ataxia; acute cerebrovascular disease; convulsions; change in sense of taste; change in sense of smell; neuralgia; myalgia; arthralgia; skin rash; vesicular lesions; maculopapules; itchy skin; and pseudo-chilblains.

### Statistical analysis

The sociodemographic variables and clinical characteristics of the sample were described using absolute frequencies and percentages. Mean and standard deviation together with minimum and maximum were used to describe the duration of symptoms. The χ² comparison was used to analyse the relationship between two categorical variables. The OR was estimated with its CI as a measure of association between the main symptoms and the PCR result, with the latter considered as the gold standard.

From the results of the bivariate analyses, logistic regressions were performed using the stepwise forward selection method to determine the best predictor symptoms, in addition to TSD, of a positive PCR result. To analyse the classification capacity of the models, the AUC was used together with the values of sensitivity, specificity, positive predictive value, and negative predictive value. The receiver operating characteristic (ROC) was used to compare and graphically represent the models.

All CIs are 95% CIs and a significance level of 5% was set. R statistical software (version 4.0.3) and SPSS (version 27) were used.

## Results

Of the 226 health professionals included, 116 (51.3%) had a positive PCR and were considered positive cases (COVID-19 positive) and 110 were considered negative cases (COVID-19 negative). The cohort was predominantly female and a significantly greater amount of COVID-19 positive participants smoked (*P* = 0.026) and were admitted more (0.002).

COVID-19 positive patients had more fever, low-grade fever, shivering, asthenia, dyspnoea, change in sense of taste and smell, arthralgia, myalgia, anorexia, nasal obstruction, sneezing, epistaxis, urticarial lesions or itching, , tachycardia, chest pain, diarrhoea, sputum production, and headache (*P*<0.005) (Table 1).

**Table 1.** Clinical characteristics of the sample, at any time

Symptom	COVID-19		COVID-19		*P*
	Positive (*n* = 116)		Negative (*n* = 110)		
	*n*	%	*n*	%	
Fever (≥38 ºC)	43	37.1	12	10.9	0.000
Febrile (<38 ºC)	81	69.8	45	40.9	0.000
Shivers	69	59.5	28	25.5	0.000
Asthenia	98	84.5	70	63.6	0.000
Dyspnoea	43	37.1	18	16.4	0.000
Alteration of taste	60	51.7	11	10.0	0.000
Alteration of smell	63	54.3	9	8,2	0.000
Arthralgias	51	44.0	19	17.3	0.000
Myalgias	82	70.7	10	9.1	0.000
Anorexia	44	37.9	20	18.2	0.001
Congestion or nasal obstruction	35	30.2	13	11.8	0.001
Sneezing	22	19.0	9	8.2	0.015
Epistaxis	8	6.9	1	0.9	0.021
Hives or itching	10	8.6	2	1.8	0.021
Instability (balance)	19	16.4	8	7.3	0.027
Tachycardia	16	13.8	6	5.5	0.028
Chest pain	23	19.8	11	10.0	0.029
Diarrhoea	52	44.8	35	31.8	0.030
Spit production	11	9.5	3	2.7	0.032
Headache	80	69.0	63	57.3	0.046

Only variables with statistically significant differences are shown.

In relation to the symptoms of COVID-19 positive patients, statistically significant differences were observed by sex; women presented more changes in sense of smell and taste, headache, and nasal obstruction.

Statistically significant differences by age group were also observed; those aged >50 years had more fever, cough, and headache, and those aged <40 years had more nasal obstruction and rhinorrhoea.

**Figure 1.**
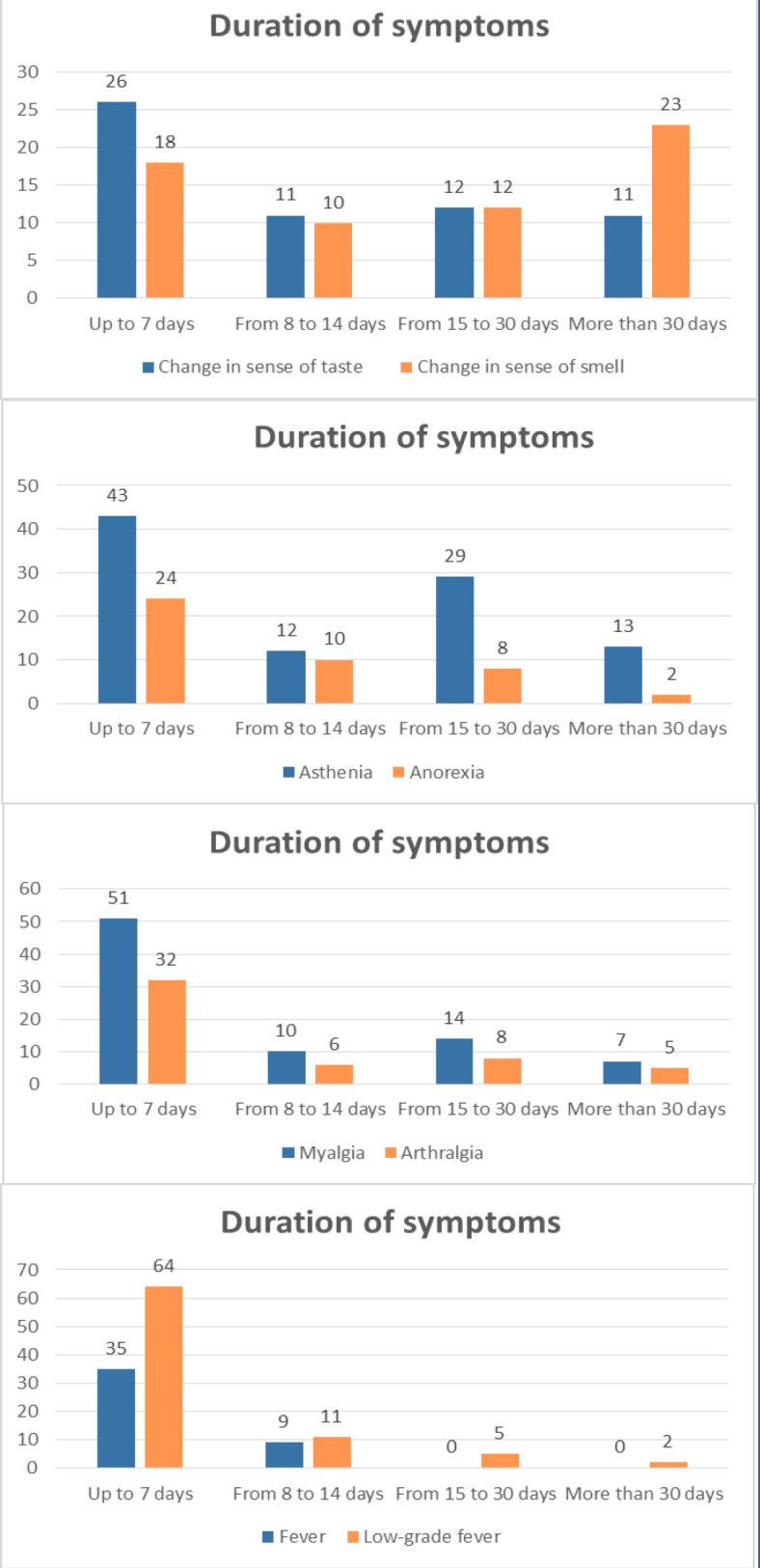
Duration of the most prevalent symptoms

Figure 1 shows the duration of the most prevalent symptoms. Symptoms with a mean duration of more than 10 days with the mean number of days of duration, standard deviation and range, are presented in a supplementary table.

**Figure 2.**
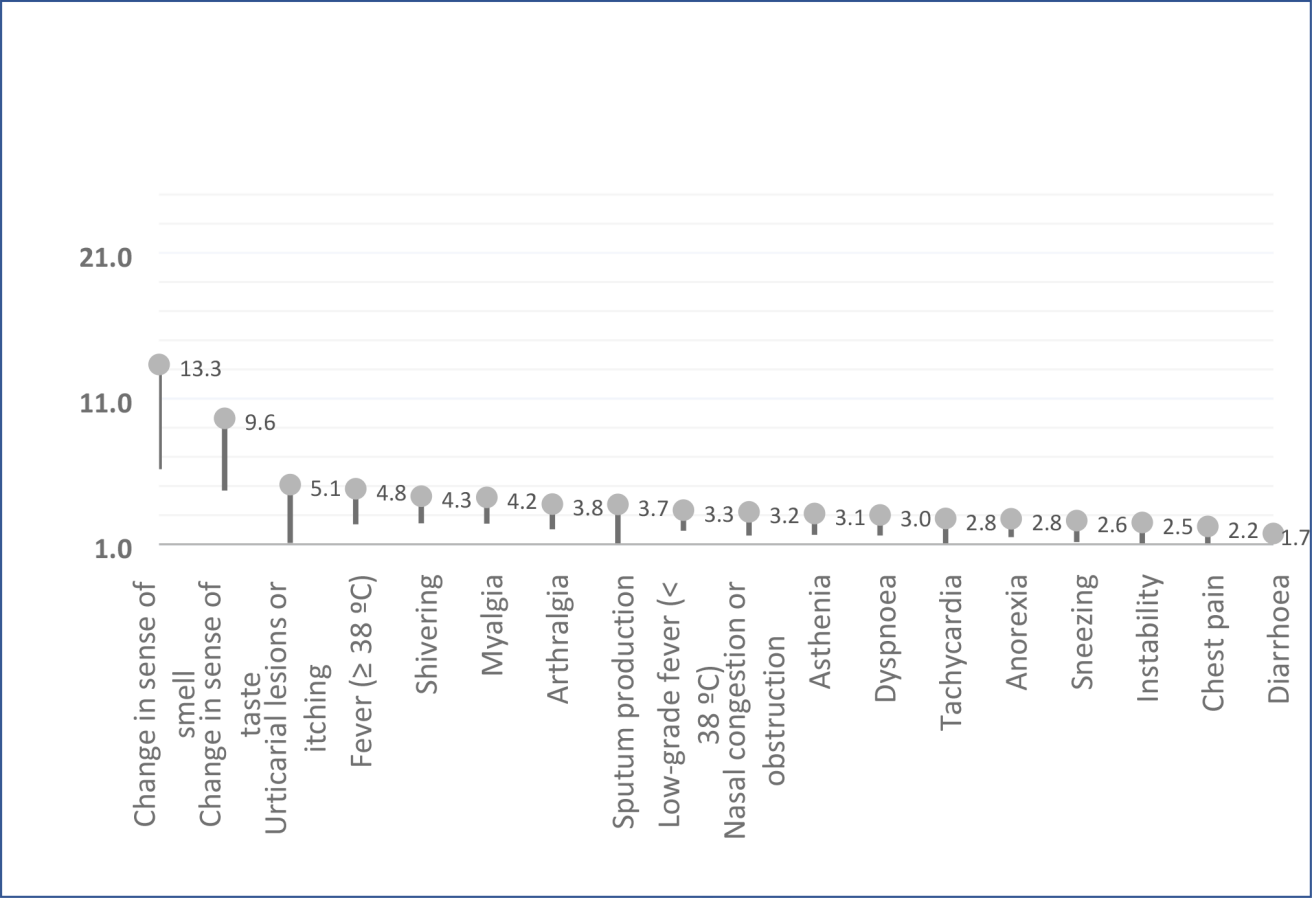
Association between symptoms and odds ratio of SARS-CoV-2 infection in 226 healthcare workers in central Catalonia who were evaluated by reverse transcription-polymerase chain reaction (RT-PCR)

Figure 2 shows the degree of association between symptoms and SARS-CoV-2 infection in the participants studied. Loss of smell stands out with an OR of 13.3 (95% CI = 6.22 to 28.9) as well as change in sense of taste with an OR of 9.6 (95% CI = 4.7 to 19.8).

Finally, with the information from the univariate descriptive and bivariate contrasts, the variables fever, low-grade fever, or shivering and the variables TSDs were grouped for logistic regression; and age, sex, TSDs, urticarial lesions or itching, temperature changes or shivering, myalgia, arthralgia, sputum production, nasal congestion, asthenia, dyspnoea, tachycardia, anorexia, sneezing, chest pain, and diarrhoea were selected as possible predictors. Using the stepwise method, the selection of the best predictors were TSDs, temperature changes or shivering, dyspnoea, myalgia, and arthralgia (Table 2). From the final model (step 5) it was observed that having fever, low-grade fever or shivering corresponded to odds almost three times higher than having a positive CRP compared with those without any temperature change or shivering. Having dyspnoea more than doubles the possibility of presenting a positive test compared with those who do not have dyspnoea; experiencing changes in sense of smell and taste corresponds to an elevenfold increase in the possibility of presenting a positive test compared with not having them; and, on the other hand, having arthralgia or having myalgia does not reach the level of significance in the logistic relationship and therefore, in this model, does not predict the positivity of the test.

**Table 2.** Classifier performance

	Predictor(s)	OR	95% CI	*P*	AUC	Senstivity	Specificity	PPV	NPV
Step 1	Changes in sense of smell or taste	12.4	(6.3 to 26.2)	<0.001	74.7%	60.3%	89.1%	85.4%	68.1%

Step 2	Changes in sense of smell or taste	10.6	(5.3 to 23.1)	<0.001	81.3%	60.3%	89.1%	85.4%	68.1%
	Fever, low-grade fever or shivering	4.6	(2.4 to 10.9)	<0.001					

Step 3	Changes in sense of smell or taste	10.2	(5.0 to 22.6)	<0.001	84.0%	86.2%	69.1%	74.6%	82.6%
	Fever, low-grade fever or shivering	3.5	(1.6 to 8.0)	0.001					
	Myalgia	2.5	(1.3 to 4.9)	0.008					

Step 4	Changes in sense of smell or taste	10.4	(5.0 to 23.0)	<0.001	84.5%	76.7%	82.7%	82.4%	77.1%
	Fever, low-grade fever or shivering	3.4	(1.6 to 7.8)	0.002					
	Myalgia	1.8	(0.8 to 3.9)	0.119					
	Arthralgia	2.1	(0.9 to 4.7)	0.082					

Step 5	Changes in sense of smell or taste	11.1	(5.3 to 25.1)	<0.001	85.7%	82.7%	80.0%	81.4%	81.5%
	Fever, low-grade fever or shivering	3.0	(1.4 to 6.8)	0.007					
	Myalgia	1.6	(0.7 to 3.5)	0.234					
	Arthralgia	2.2	(1.0 to 5.1)	0.065					
	Dyspnoea	2.4	(1.1 to 5.3)	0.030					

AUC = area under the curve. NPV = negative predictive value. PPV = positive predictive value.

**Figure 3.**
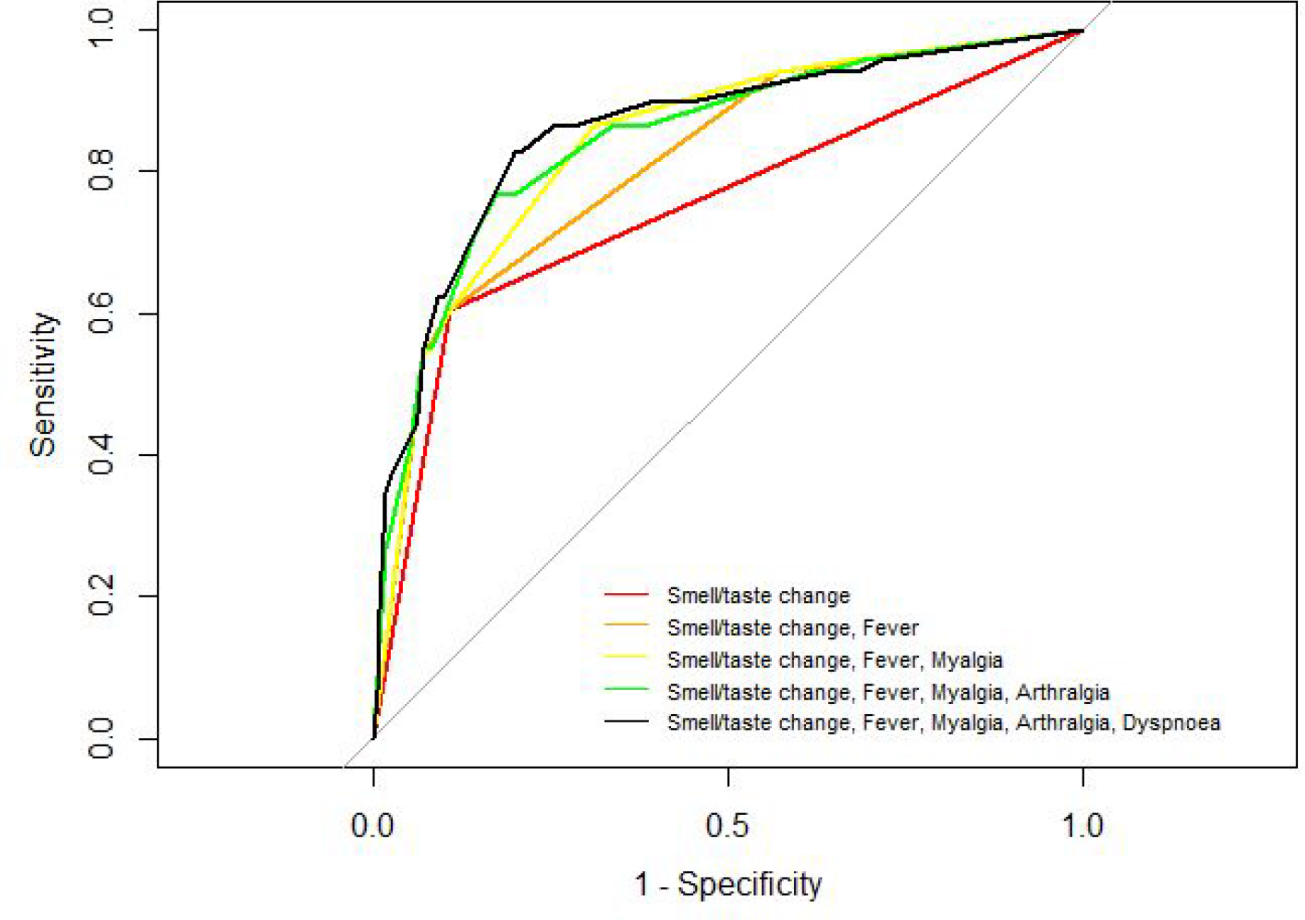
Receiver operating characteristic (ROC) plots for symptom classifier models. The dashed diagonal line shows a non-diagnostic result. Area under the curve (AUC) for each symptom classifier group is displayed in Table 2. *(A non-colour dependent version of this figure is available in the supplementary materials)*

Regarding the discriminative capacity of the models, Figure 3 shows the ROC curves of the variable TSDs and the curves of progressively adding the rest of the selected variables according to the importance in the model and the clinical criterion. The final model has an AUC of 85.7% (95% CI = 80.7% to 90.7%), with a sensitivity of 82.8%, a specificity of 80.0%, a positive predictive value of 81.4%, and a negative predictive value of 81.5%.

## Discussion

### Summary

TSDs are a strong predictor of a positive COVID-19 result. In the study, the association of TSD, fever or low-grade fever, shivering, dyspnoea, arthralgia, and myalgia correctly predicted 85.7% of the results of the COVID-19 test. Further studies are needed to confirm the potential of this association.

### Strengths and limitations

The main limitation is the fact that it is a retrospective study, meaning some mild symptoms may have gone unnoticed and participants may have had difficulty in remembering dates.

The authors of this study did not contemplate the use of specific TSD tests for chemosensory assessment, even though such tests would have been ideal. Regarding benefit–risk balance, its use was considered to involve unnecessary additional time and risk of exposure to COVID-19 by clinicians, as well as non-essential discomfort for patients.

Finally, the sensitivity and potential false negativity of PCR-based COVID-19 testing must also be considered.

One of the strengths of the study is that it was conducted on health professionals who are knowledgeable regarding the symptoms presented, and through telephone interviews. Other published studies have involved the general population, anonymously and self-reported through smartphone applications.^13–16^

### Comparison with existing literature

The study aimed to evaluate the symptoms associated with a positive COVID-19 test. TSDs in the COVID-19 positive study sample appeared in similar proportions to 35 other European studies with 8575 patients, with a prevalence of 57.5% of olfactory dysfunction and 53.1% of taste dysfunction.^17^

In the sample, patients with severe hyposmia or anosmia usually also complained of loss of taste, as also reported by most articles included in the systematic review and meta-analysis of Ibekwe *et al*.^18^ This is owing to loss in the contribution of smell to their perception of flavour. Paderno *et al* ^19^ observed that TSDs were more frequent in participants with mild-to-moderate COVID-19 who did not require admission. The recovery of olfactory and gustatory functions also varies according to studies. In the present study, 61.1% had regained their sense of taste and 44.4% had recovered their sense of smell 14 days after the onset of clinical symptoms. In Barillari *et al*’s study, 24.3% of patients regained their sense of smell and taste within 9–15 days.^9^ And in Lechien *et al*’s study, 25.5% of patients regained these functions within 2 weeks after resolution of general symptoms.^8^

Regarding sex differences, women presented more clinical symptoms of headache, TSD, and nasal obstruction. Other studies agree that the incidence of TSDs is higher in women than in men.^20–22^ Women are more likely to develop post-infectious olfactory dysfunction in viral infections related to parainfluenza or Epstein-Barr virus.^10^ Looking at age, it is notable that younger patients more often present ear, nose, and throat (ENT) clinical symptoms (nasal obstruction and rhinorrhoea), and these data coincide with the Italian studies of Barillari *et al* and Lechien *et al,* ^8,9,23^ which also found a negative correlation between ENT symptoms and age.

One meta-analysis confirmed that the prevalence rates of olfactory and gustatory dysfunction were different among four geographical regions of the world. The prevalence of olfactory dysfunction in East Asia was significantly lower than that in Europe or the Middle East, and prevalence of gustatory dysfunction in East Asia was significantly lower than that in Europe and North America.^17^ A hypothesis proposed by Li *et al* ^24^ and Forster *et al* ^25^ in relation to the differences between both regions of the world (East Asia, and Europe and North America), on olfactory and taste dysfunctions, indicated that this could be related to differences in the genetic pattern of the virus (potential mutations). Another hypothesis indicated that the variation can be related to angiotensin-converting enzyme 2 (ACE2), a possible host receptor of SARS-CoV-2.^26,27^ The presence of a difference in variants of ACE2 according to geographical and ethnic factors has been demonstrated.^28^ It is assumed that the difference in variants of ACE2 expressed in olfactory epithelial cells, according to populations from different geographical regions, can influence the prevalence of olfactory and gustatory dysfunction.^17^

TSDs were the symptoms with the strongest association for a positive COVID-19 result, with an OR of 13.3 for loss of sense of smell and 9.6 for loss of taste. Overall, TSDs had an OR of 12.4, a sensitivity of 60.3%, and a specificity of 89.1%. These results coincide with Menni’s study, which had a sample of US and UK patients.

The ability of the symptom set to accurately classify subjects as COVID-19 positive was identifed and assessed through logistic regression. Symptoms associated with COVID-19 positivity included TSDs, fever or low-grade fever, shivering, dyspnoea, arthralgia, and myalgia, with an AUC of 85.7% (95% CI = 80.7% to 90.7%). The results are in broad agreement with other studies but with differences: Roland *et al* ^13^ also identified odynophagia; Menni *et al* ^15^ cough and digestive clinical signs; and the results of Yan *et al* ^14^ showed no significance for dyspnoea.

### Implications for practice

The findings of this study suggest that TSDs could be included as part of routine screening for COVID-19. These symptoms could be used in mass screening by professionals with limited medical knowledge and using telemedicine. These data could be useful where diagnostic testing for COVID-19 in the general population is difficult and/or in situations with high patient volume and diagnostic testing difficulties. The fact that COVID-19 can be diagnosed without the need for PCR allows early diagnosis and isolation. Future research may be needed in relation to novel or emerging strains.

## Data Availability Statement:

The data presented in this study are available on request from the corresponding author.
